# Toward high-fidelity coherent electron spin transport in a GaAs double quantum dot

**DOI:** 10.1038/s41598-018-31879-4

**Published:** 2018-09-18

**Authors:** Xinyu Zhao, Xuedong Hu

**Affiliations:** Department of Physics, University at Buffalo, SUNY, Buffalo, New York, 14260-1500 USA

## Abstract

In this paper, we investigate how to achieve high-fidelity electron spin transport in a GaAs double quantum dot. Our study examines fidelity loss in spin transport from multiple perspectives. We first study incoherent fidelity loss due to hyperfine and spin-orbit interaction. We calculate fidelity loss due to the random Overhauser field from hyperfine interaction, and spin relaxation rate due to spin-orbit interaction in a wide range of experimental parameters with a focus on the occurrence of spin hot spots. A safe parameter regime is identified in order to avoid these spin hot spots. We then analyze systematic errors due to non-adiabatic transitions in the Landau-Zener process of sweeping the interdot detuning, and propose a scheme to take advantage of possible Landau-Zener-Stückelberg interference to achieve high-fidelity spin transport at a higher speed. At last, we study another systematic error caused by the correction to the electron *g*-factor from the double dot potential, which can lead to a notable phase error. In all, our results should provide a useful guidance for future experiments on coherent electron spin transport.

## Introduction

In universal quantum computing, quantum information inevitably needs to be transferred over finite distances on chip or between chips. For spin qubits in semiconductor nanostructures^1–9^, there are a variety of ways such long-distance communication can be achieved^10–17^. One particularly straightforward way is to move the electrons themselves between quantum dots. Indeed, coherently transporting electrons between quantum confined states, with their spin states intact, could be a critical component of a wide range of future quantum coherent devices that utilize the electron spins.

There are two major approaches to achieve coherent transport of spin qubits, one using surface acoustic waves^10,18–26^. the other by tuning the electric potentials on a series of surface gates^11,12,27–31^. We have studied the former in the past^22,25^, and will in this paper focus on the latter, which is an integral part of a concerted experimental effort towards making larger arrays of quantum dots^30–32^. Indeed, the importance of coherent spin transport goes well beyond quantum information transfer. Other important quantum operations, such as error correction and spin readout, also involve electron tunneling between quantum dots^30,33–37^. In the broader context of semiconductor heterostructures, an investigation of transport properties between quantum dots and nanowires is also an important element in the search and control of possible Majorana fermion excitations^38,39^.

Practically, quantum tunneling of an electron is usually driven by tuning the bias voltage between neighboring quantum dots. During such a process, several factors could change the spin state of the electron and reduce the fidelity of spin transfer. A recent paper has already investigated the intrinsic errors in several aspects in the transport^40^, such as preparing the initial state, gate operations, and finalization. In this paper, we model the initialization, the transport, and the finalization as a continuous dynamical process, and study several external factors that can impact this process. For example, spin relaxation due to spin-orbit interaction (SOI)^41,42^ and phonon emission could be modified by the double-dot confinement as opposed to a single-dot confinement^43^. The degeneracy near zero bias causes an energy level anti-crossing, so that a time-dependent Hamiltonian for sweeping the electric field with a finite speed could cause non-adiabatic transitions, which usually reduce the fidelity of the electron spin transfer. Furthermore, the SOI together with the confinement potential causes corrections to the eigen-energies, leading to modification of the effective *g*-factor, which could be significant if a superposed spin state is being transferred.

In this work, we study how to achieve high-fidelity spin and charge transfer through electron tunneling in a double dot. We first quantitatively study spin decoherence caused by hyperfine interaction and SOI to ensure that there is no significant fidelity loss due to these incoherent processes. We explicitly calculate the residue coherence after the transport when taking spin relaxation and dephasing effects into consideration. After clarifying the decoherence errors caused by interaction with external environments, we analyze several systematic errors in the electron transport. In particular, we show that at finite sweeping speed for the interdot detuning, Landau-Zener (LZ) process leads to unwanted spin transitions that lower the spin transfer fidelity. We then show how pulse shaping can help reduce this population error by suppressing the LZ process. We also propose a scheme to achieve high-speed and high-fidelity electron transport through Landau-Zener-Stückelberg (LZS) interference, which can also be used to measure the tunnel barrier between the two dots. Last but not least, we study an important correction to the effective *g*-factor by SOI and the double dot potential. We point out that this correction can cause a significant error in the tracking of the phase difference between spin up and down states, and needs to be properly accounted for by mapping out the system parameters accurately during the detuning sweeping process.

## Results

**Figure 1:**
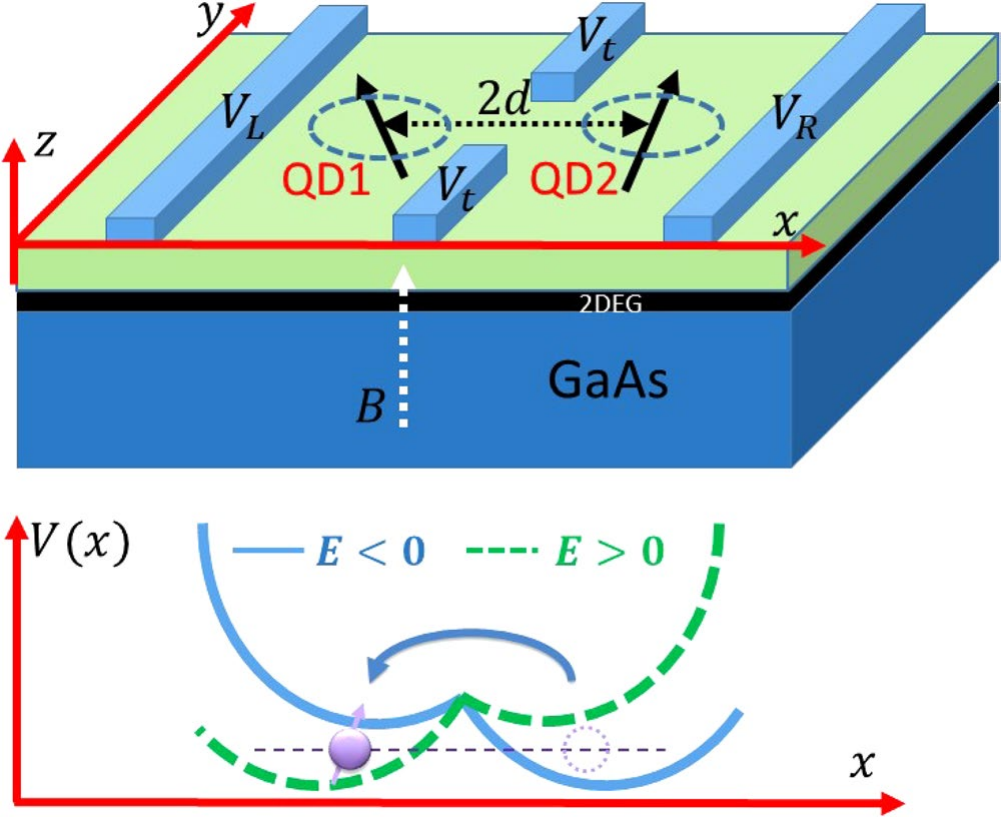
Sketch of our protocol for electron transport in a double quantum dot. The two-dimensional DQD resides at the interface of GaAs and the barrier material, with the growth-direction confinement much stronger than the in-plane confinement. The regions “QD1” and “QD2” label the two dots. Surface gates *V*_*L*_ and *V*_*R*_ can be used to adjust the detuning between the two dots, while *V*_*t*_ can be used to tune the tunnel coupling strength.

In this paper we study electron spin transport that is enabled by tuning the applied voltages on the metallic surface gates. While a dense array of gates together with optimized programming of voltages can probably achieve relatively smooth motion of a quantum dot potential, here we focus on a much simpler protocol. Assuming the existence of a double quantum dot (DQD) potential, as illustrated in Fig. 1, changing the interdot detuning via an applied electric field shifts the ground orbital state from one dot to the other, thereby achieving electron transport. In such a process, the only time-dependent variable is the electric field applied across the DQD, tunable by one or two surface gates.

The goal of our protocol is to transfer the complete spin information from one dot to the other quickly and faithfully. This transfer entails the transfer of both the carrier itself, i.e. the electron, and the spin state. Obviously, multiple factors could affect the fidelity of this transfer, which we study in this work.

We first focus on incoherent errors in spin transfer due to spin decoherence. In GaAs, both the hyperfine interaction and the SOI could cause spin decoherence. Here we estimate rates of spin relaxation and dephasing caused by both factors, and examine fidelity loss in the transport due to these incoherent processes. We study the conditions under which spin relaxation and dephasing can cause notable errors, and discuss how to avoid or suppress these errors.

After identifying regimes where incoherent errors should be negligible, we examine systematic errors due to our protocol. Specifically, our protocol is performed non-adiabatically in general. With a finite speed for transport that we would like to push as fast as we can, the electron would have a non-vanishing probability of being excited to higher orbital and/or spin states, which reduces spin transfer fidelity. Here we study these non-adiabatic transitions and propose possible schemes to enhance the transport fidelity, by either weakening these transitions or using interference to suppress their net effect.

Another important systematic error originates from modification to the electron energy spectrum as it is being transferred in a double quantum dot. In particular, near zero-detuning between the double dot, the electron spin splitting would be different from that in a single dot because of the different confinement. This SOI-induced correction modifies the electron *g*-factor, and introduces an additional dynamical phase between different spin states when a superposition state is transported. We will show in the last subsection that this phase error could be significant, so that one has to keep track of this phase change in order to maintain the correct superposition.

### Model of a double quantum dot

The system we consider is a two-dimensional GaAs DQD with an electric field applied along the interdot axis. The confinement along the growth direction is much stronger so that we do not consider any excitation in that direction. The system Hamiltonian is thus given by

1 $$H=T+{V}_{0}+{H}_{E}+{H}_{Z}+{H}_{SO}+{H}_{hf},$$

where

2 $$T=\frac{{\pi }^{2}}{2{m}^{\ast }},$$

3 $${V}_{0}(x,y)=\frac{1}{2}{m}^{\ast }{\omega }_{0}^{2}[{(|x|-d)}^{2}+{y}^{2}],$$

4 $${H}_{E}=eEx,$$

5 $${H}_{Z}=\frac{1}{2}g{\mu }_{B}B{\sigma }_{z},$$

6 $${H}_{SO}=\frac{{\alpha }_{BR}}{\hslash }({\sigma }_{x}{\pi }_{y}-{\sigma }_{y}{\pi }_{x})+\frac{{\alpha }_{D}}{\hslash }({\sigma }_{y}{\pi }_{y}-{\sigma }_{x}{\pi }_{x}),$$

7 $${H}_{hf}=\frac{1}{2}g{\mu }_{B}{{\bf{B}}}_{nuc}\cdot \sigma .$$

Here *π* = **p** + *e***A** is the kinetic momentum operator, m* the effective mass of the electron, *e* the absolute value of electron charge, and **A** = *B*(−*y*/2, *x*/2, 0) is the vector potential of the applied magnetic field. The external magnetic field is applied along the *z*-direction (growth direction), which introduces a Zeeman splitting given by *H*_*Z*_. The DQD confinement potential is modeled by a double harmonic *V*_0_^43–45^, where *d* gives the half interdot distance. In this simple model, varying the interdot distance also changes the tunnel barrier between the two dots. The interdot detuning *V*_*d*_ = 2*eEd* is controlled by an electric field via *H*_*E*_, which in practice can be tuned by voltages applied on gates *V*_*L*_ and *V*_*R*_, as shown in Fig. 1. *V*_0_ and *H*_*E*_ together gives the total electric potential *V* = *V*_0_ + *H*_*E*_, which is schematically plotted in the bottom panel of Fig. 1 in two cases: *E* < 0 (blue solid line) and *E* > 0 (green dashed line). The electron transport is achieved by tuning the electric field *E*. In other words in our protocol *E* = *E*(*t*). The time dependen*t* electric field *E*(*t*) also produces an ex*t*ra time dependent magnetic field *B*(*t*) since ${\rm{\nabla }}\times {\bf{B}}=\frac{1}{{c}^{2}}\frac{{\rm{\partial }}E(t)}{{\rm{\partial }}t}$. However, the amplitude *B*(*t*) is extremely small (proportional to 1/*c*), as expected as a relativistic correction^46^. Therefore, we neglect the effect of *B*(*t*).

Lastly, *H*_*SO*_ and *H*_*hf*_ describe two major mechanisms of spin mixing. *H*_*SO*_ is the spin-orbit coupling, where *α*_*D*_ and *α*_*BR*_ are the strength of Dresselhaus and Bychkov-Rashba SOI, respectively^41–43,47^. *H*_*hf*_ is the hyperfine interaction between the electron and the environmental nuclear spins. In our calculation we take the mean-field approximation for the nuclear spins, so that their effect is modeled as an effective magnetic field **B**_*nuc*_, the Overhauser field. Under normal experimental conditions, the Overhauser field is in an arbitrary direction and is position-dependent. Generally the longitudinal component of **B**_*nuc*_ (parallel to the external magnetic field) causes a small modification of the Zeeman energy, and the transverse components make spin-flip transitions possible.

Our numerical results in this paper are obtained using the following parameters unless otherwise noted. The half inter dot distance is chosen as *d* = 46 nm, the confinement energy of single dot is ℏ*ω*_0_ = 1.1 meV, the bulk value of *g*-factor is *g* = −0.44, the effective mass of the electron is m* = 0.067*m* = 6.097 × 10^−32^ kg, and the Dresselhaus and Bychkov-Rashba SOI strength are *α*_*D*_ = 4.5 meV · Å and *α*_*BR*_ = 3.3 meV · Å^48^.

To obtain spin transfer fidelity in our protocol, we solve the time evolution of the electron state governed by the time-dependent Schrödinger equation in the basis {*x*, *y*, *σ*}

8 $$i\hslash \frac{\partial }{\partial t}|\psi (x,y,\sigma ,t)\rangle =H(t)|\psi (x,y,\sigma ,t)\rangle ,$$

where *σ* indicates the spin states. We also solve the instantaneous eigenstates |*ψ*_*i*_(*t*)〉 and eigenenergies *ε*_*i*_(*t*) by numerically diagonalizing the Hamiltonian *H*(*t*) at an electric field *E*(*t*) for a series of points in time. Then, the state obtained in Eq. (8) can be expanded as $|\psi (x,y,\sigma ,t)\rangle ={\sum }_{i}\,{C}_{i}(t)|{\psi }_{i}(x,y,\sigma ,t)\rangle$. With *C*_*i*_(*t*), one can monitor to what extent the state follows an adiabatic evolution. In the numerical simulation, the lowest twenty eigenstates are included. The full numerical simulations in this paper is a complementary to the previous research^40^ based on the analysis of 4 × 4 Hamiltonian with lowest orbital level.

**Figure 2:**
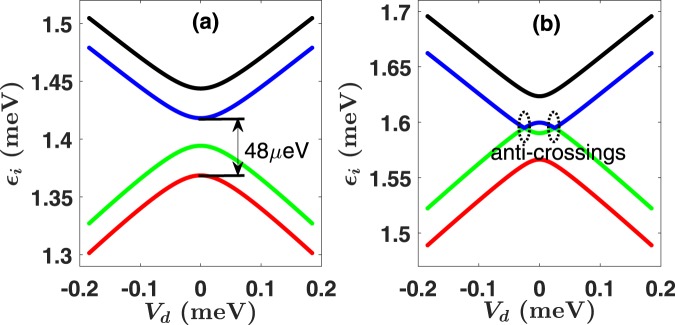
Typical energy diagrams for a DQD. The interdot detuning is given by *V*_*d*_ = 2*eEd*. The magnetic field is *B* = 1 T for (**a**) and *B* = 1.3 T for (**b**). In panel (a), from top to bottom, the black, blue, green, and red lines represent the energy levels of the states $|e,\downarrow \rangle$, $|e,\uparrow \rangle$, $|g,\downarrow \rangle$, and $|g,\uparrow \rangle$. For *B* = 1.3 T, when Zeeman energy is larger than the tunnel coupling, the energy of $|e,\uparrow \rangle$ is smaller than the energy of $|g,\downarrow \rangle$ near *V*_*d*_ = 0.

In Fig. 2(a) we plot a typical low-energy diagram *ε*_*i*_(*t*) of the DQD. When spin-orbit mixing is negligible, from top to bottom, the four curves represent the energy levels of the states $|e,\downarrow \rangle$, $|e,\uparrow \rangle$, $|g,\downarrow \rangle$, and $|g,\uparrow \rangle$, where |*g*〉 is the ground orbital state and |*e*〉 is the first excited orbital state, $|\uparrow \rangle$ and $|\downarrow \rangle$ indicate the spin states. Essentially each orbital state splits into two roughly parallel spin branches. When ${V}_{d}\ll -\,{t}_{E}$ (*t*_*E*_ is tunneling barrier, i.e., the half energy gap at zero detuning), the ground orbital state |*g*〉 is approximately the lowest-energy Fock-Darwin state located in the right dot |*ψ*_*R*_〉 ∝ exp{[−(*x* − *d*)^2^ − *y*^2^]/2*a*^2^}, and the excited state |*e*〉 is approximately the ground Fock-Darwin state located in the left dot |*ψ*_*L*_〉 ∝ exp{[−(*x* + *d*)^2^ − *y*^2^]/2*a*^2^}, where $a={(\hslash /{m}^{\ast }\sqrt{{\omega }_{0}^{2}+{\omega }_{c}^{2}/4})}^{1/2}$ is the effective confinement length, with *ω*_*c*_ = *eB*/m*. When ${V}_{d}\gg {t}_{E}$, the ground state and excited states are switched, and the left dot Fock-Darwin state |*ψ*_*L*_〉 becomes the ground state. Near the zero detuning *V*_*d*_ = 0, |*g*〉 and |*e*〉 are mixtures of |*ψ*_*L*_〉 and |*ψ*_*R*_〉, and an anti-crossing forms with an energy gap 2*t*_*E*_. This makes our protocol essentially a LZ process, which will be analyzed in detail in the following sections.

With the correction of Zeeman energy, the spin-up excited state $|e,\uparrow \rangle$ could have equal or even lower energy than the state $|g,\downarrow \rangle$ near *V*_*d*_ = 0 when the magnetic field is above the threshold given by the tunnel coupling. An example is given in Fig. 2(b) for a relatively large *B* field. Near zero detuning, two anti-crossings between different spin states are formed. Through SOI, spin states are mixed near the two anti-crossings in Fig. 2(b), which allow transitions between eigenstates of the far-detuned limit. We will discuss the consequences of these anti-crossings in the following subsections.

### Spin transfer fidelity loss due to incoherent processes

Spin decoherence in quantum dots generally involve two major interactions: spin dephasing due to the random longitudinal Overhauser field from the hyperfine interaction, and spin relaxation due to spin mixing from SOI (or hyperfine interaction), and electron-phonon interaction to facilitate transitions between states with different energies. In this subsection, we calculate the rate of spin relaxation rates when either hyperfine interaction or SOI is solely taken into consideration, and show that relaxation can be neglected if experimental parameters are chosen properly.

#### Fidelity loss due to hyperfine interaction

We first investigate fidelity loss caused by hyperfine interaction^49–51^. As discussed in the model Hamiltonian subsection, we treat hyperfine interaction within the mean field approximation, so that nuclear spin effects are fully represented by the Overhauser field **B**_*nuc*_. This approximation is justified by the vastly different time scales involved in our study: on the one hand, electron tunneling in the double dot happens at the nanosecond time scale; on the other hand, nuclear spin precession time is in the order of microseconds, while nuclear spin diffusion happens at even longer time scale of milliseconds to seconds. The much longer time scale for the nuclear spins mean that within a tunneling experiment of a few nanoseconds, the nuclear spins are essentially static. Their effects are spatially disordered but temporally constant at the lowest order approximation.

The longitudinal part of **B**_*nuc*_, the *z* component, causes inhomogeneous broadening for the electron spin^52^. The pure dephasing caused by the quasi-static nuclear field can be effectively eliminated by spin-echo or other dynamical decoupling schemes^52–54^. Besides, electron motion allows the electron spin to sample more nuclear spins, therefore reducing their dephasing effect via motional narrowing, as discussed in ref.^22^.

**Figure 3:**
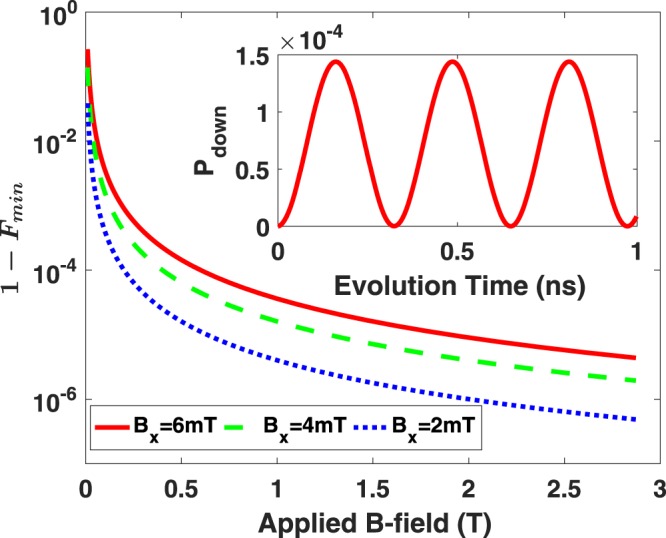
Spin fidelity loss caused by hyperfine interaction. The main figure shows the largest deviation from the initial state during the whole evolution. The inset is a dynamical evolution in 1 ns of the spin for the parameters *B* = 0.5 T, *B*_*nuc*,*x*_ = 6 mT.

The transverse part of the Overhauser field slightly tilts the quantization axis for the electron spin, and causes a spin in the original eigenstate to precess (this is equivalent to the picture of spin mixing due to the transverse Overhauser field). Consider for instance a nuclear field with only an *x*-component. The electron spin Hamiltonian is then ${H}_{spin}=\frac{1}{2}g{\mu }_{B}(B{\sigma }_{z}+{B}_{nuc,x}{\sigma }_{x})$ (assuming no SOI, so that orbital part is decoupled). If the electron spin is initially prepared in a spin up state ($|\uparrow \rangle$ in the *z*-direction of the lab frame), the evolution of the spin would then simply be a precession with respect to the new quantization axis that differs slightly from the *z*-axis due to the nuclear spin correction, $|{\psi }_{spin}(t)\rangle =\exp (\,-\,i{H}_{spin}t)|\uparrow \rangle$. As the spin precesses, it periodically deviates from the *z*-direction, as shown in the inset of Fig. 3. The minimum fidelity ${F}_{{\min }}=\,{\rm{\min }}\{|\langle {\psi }_{spin}(t)|\uparrow \rangle {|}^{2}\}$ is (when the spin has the largest deviation from the original eigenstate during the evolution)

9 $${F}_{{\min }}=\frac{{B}^{2}}{{B}^{2}+{B}_{nuc,x}^{2}}.$$

In a typical GaAs quantum dot, *B*_*nuc*_ (or *B*_*nuc*,*x*_) ~ 2–6 mT^51^, while the external field *B* is typically much larger, around *B* = 1 T. Equation (9) would give a fidelity of 0.9999 if *B* is about 100 times larger than *B*_*nuc*,*x*_. Therefore, the fidelity loss caused by the transverse part of the Overhauser field should be negligible under appropriate experimental conditions before electron tunneling is taken into consideration.

When electron tunnels from one dot to another, the nuclear environment changes and the Overhauser field would generally be different and uncorrelated between the two dots. While motional narrowing would help reduce inhomogeneous broadening from the longitudinal Overhauser field^22^ (although in a double dot this averaging effect is still not prominent because we are adding only two random numbers), it should also help reduce the effect of the transverse Overhauser field, as the quantization axis would be tilted in a different direction when the electron moves from one dot to the other.

In ref.^55^, the authors also studied the correction on electric dipole matrix elements caused by the nuclear spins. Similarly, they also showed that nuclear spins only cause a small correction to the relaxation rate in the case |**B**_*nuc*_| = 3 mT.

In summary, we find that while hyperfine interaction can negatively impact the spin transfer fidelity in our protocol, its effects can be suppressed by optimizing the experimental conditions and applying correctional measures such as spin echoes. In particular, electron spin experiences a strong inhomogeneous broadening and the associated dephasing due to the longitudinal Overhauser field. Transport can actually help reduce this effect because of motional narrowing, although spin echo or other dynamical decoupling pulses would be needed for high-fidelity spin transfer. Transverse Overhauser field, on the other hand, causes a tilting of the electron spin quantization axis and leads to spin mixing. However, by applying an external field that is sufficiently large compared to the Overhauser field (for example, 1 T in GaAs, where random Overhauser field is in the order of a millitesla), the axis tilting effect is strongly suppressed. Transport would lead to further suppression of this effect via motional narrowing, so that the effect of the transverse Overhauser field should be negligible in general.

#### Spin-orbit interaction induced spin relaxation

In this subsection, we focus on the relaxation caused by SOI. As shown in multiple previous studies, spin relaxation due to spin-orbit coupling and phonon emission is usually the most important spin relaxation mechanism for a quantum dot confined electron spin in GaAs^48,55–59^. When the electron is being transported with a constant velocity, Doppler effect causes modifications to the spin relaxation rate and angular distribution of the emitted phonons^22,25,58^. However, in the present case of an electron moving in a double dot, the speed of motion is quite slow and the Doppler shift is negligible. Our focus is thus more on how interdot coupling may modify the spin-phonon coupling and spin relaxation under quasi-static condition, and the transition rates we calculate are between instantaneous eigenstates.

Spin mixing is already included in our calculation of the instantaneous eigenstates when we diagonalize Hamiltonian (1) that contains SOI. For electron-phonon interaction we consider both deformation potential and piezoelectric interaction between the confined electron and the acoustic phonon environment. The interaction Hamiltonians are

10 $${H}_{df}={\Xi }_{d}\,\sum _{{\bf{k}}}\,\sqrt{\frac{\hslash |{\bf{k}}|}{2\rho V{c}_{1}}}{e}^{i{\bf{k}}\cdot {\bf{r}}}({b}_{{\bf{k}}\mathrm{,1}}+{b}_{-{\bf{k}}\mathrm{,1}}^{\dagger }),$$

11 $${H}_{pz}=-\,i{h}_{14}\,\sum _{{\bf{k}},\lambda }\,\sqrt{\frac{\hslash }{2\rho V{c}_{\lambda }|{\bf{k}}|}}{M}_{\lambda }{e}^{i{\bf{k}}\cdot {\bf{r}}}({b}_{{\bf{k}},\lambda }+{b}_{-{\bf{k}},\lambda }^{\dagger }\mathrm{)}.$$

Here *λ* = 1, 2, 3 indicates phonon polarization (1 for the longitudinal mode, while 2 and 3 for the two transverse modes), **k** = (*k*_*x*_, *k*_*y*_, *k*_*z*_) is the phonon wave vector, *Ξ*_*d*_ = 7 eV is the GaAs deformation potential, *h*_14_ = 1.4 × 10^9^ eV/m is the piezoelectric constant, *ρ* = 5.3 × 10^3^ *kg*/*m*^3^ is the mass density, *c*_1_ = 5.3 × 10^3^ *m*/*s* and *c*_2_ = *c*_3_ = 2.5 × 10^3^ *m*/*s* are the speeds of sound for longitudinal and transverse phonons in bulk GaAs, and *b*_*k*,*λ*_ and ${b}_{k,\lambda }^{\dagger }$ are the annihilation and creation operators for phonons in mode *λ* and with wave vector **k**. The piezoelectric interaction matrix element is ${M}_{\lambda }=2({k}_{x}{k}_{y}{e}_{z}^{\lambda }+{k}_{z}{k}_{x}{e}_{y}^{\lambda }+{k}_{y}{k}_{z}{e}_{x}^{\lambda })$, where ${e}_{x}^{\lambda }$, ${e}_{y}^{\lambda }$, ${e}_{z}^{\lambda }$ are the components of the unit polarization vectors^48^.

Given the electron-phonon interaction Hamiltonian and the electron eigenstates, the relaxation rate between two eigenstates can be computed by Fermi’s golden rule as

12 $${{\rm{\Gamma }}}_{df}=[\bar{n}+1]\frac{{\Xi }_{d}^{2}{\varepsilon }_{fi}^{2}}{8{\pi }^{2}\rho {c}_{1}^{4}{\hslash }^{3}}\,\int \,{d}^{2}{\bf{k}}|\langle {\psi }_{f}|{e}^{i{\bf{k}}\cdot {\bf{r}}}|{\psi }_{i}\rangle {|}^{2}/{k}_{z}^{1}$$

13 $${{\rm{\Gamma }}}_{pz}=[\bar{n}+1]\,\sum _{\lambda }\,\frac{{({h}_{14})}^{2}}{8{\pi }^{2}\hslash \rho {c}_{\lambda }^{2}}\,\int \,{d}^{2}{\bf{k}}|{M}_{\lambda }{|}^{2}|\langle {\psi }_{f}|{e}^{i{\bf{k}}\cdot {\bf{r}}}|{\psi }_{i}\rangle {|}^{2}/{k}_{z}^{\lambda }$$

where *ε*_*fi*_ is the energy difference between the initial and final state (|*ψ*_*i*_〉 and |*ψ*_*f*_〉), and $\bar{n}$ is the thermal occupation number of the phonon state at the energy *ε*_*fi*_, which is approximately zero for most Zeeman splitting at the dilution fridge temperature (*T* ≈ 0).

The total relaxation rate Γ = Γ_*df*_ + Γ_*pz*_ gives the transition probability $|{\psi }_{i}\rangle =|g,\downarrow \rangle \to |{\psi }_{f}\rangle =|g,\uparrow \rangle$, which is an amplitude damping channel^60^ with damping rate Γ(*t*). Such a process can be described by the “quantum jump” approach^61^ or the equivalent master equation shown in section “Method”. A formal solution for the off-diagonal elements of the reduced density matrix *ρ*(*t*) for spin only is

14 $${\rho }_{12}(t)={\rho }_{12}(0)\,\exp \,[-ig{\mu }_{B}B-{\int }_{0}^{t}\,ds\frac{{\rm{\Gamma }}(s)}{2}].$$

**Figure 4:**
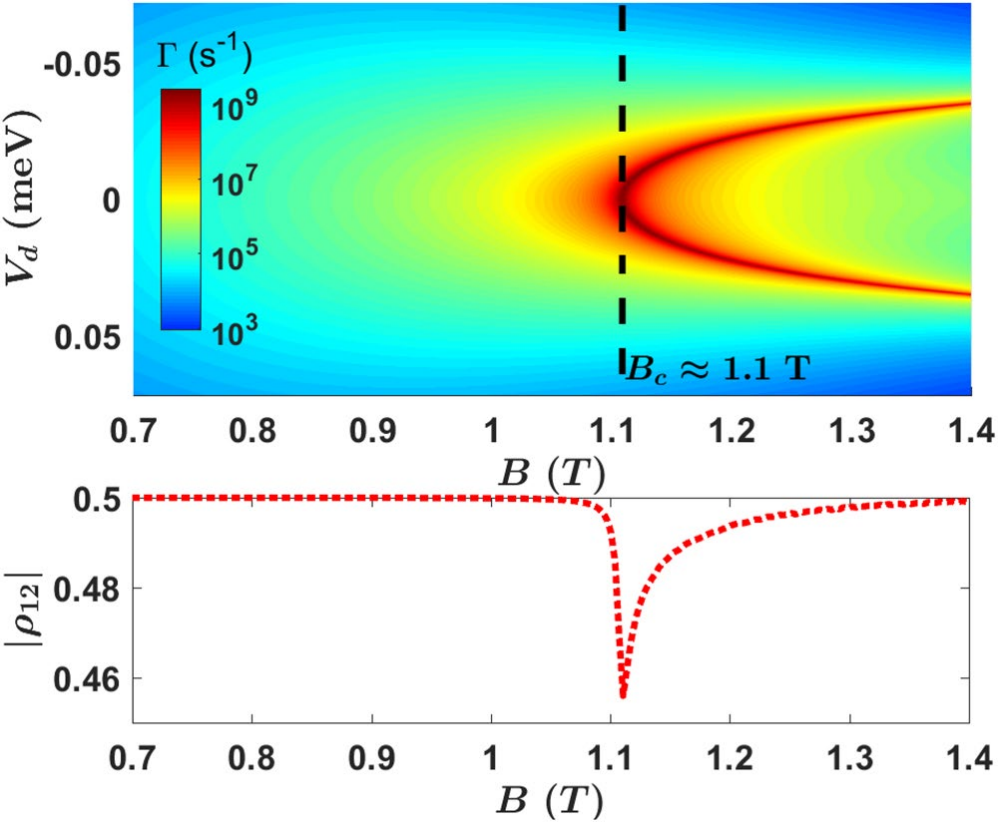
Upper panel: Spin relaxation rate as a function of the applied electric and magnetic field. Interdot detuning *V*_*d*_ = 2*eEd* with *d* = 48 nm. Lower panel: The actual residue coherence after the transport in 1 ns.

In general, electron spin relaxation rate depends on the applied electric and magnetic fields. In Fig. 4 we plot the overall spin relaxation rate Γ = Γ_*df*_ + Γ_*pz*_ as a function of both *E*- and *B*-field. The most prominent features are the sharp peaks for the relaxation rate, which are called spin hot spots^48,55,59,62^. The relaxation rate at these peaks are in the order of GHz, on par with a normal charge qubit. These hot spots are produced by the SOI-induced anti-crossing and mixing between states $|g,\downarrow \rangle$ and $|e,\uparrow \rangle$. At these anti-crossings spin is not a good quantum number, so that the relaxation rate is determined by the charge relaxation matrix element between |*g*〉 and |*e*〉.

The electric- and magnetic-field dependence of the hot spots are straightforward. In a low magnetic field, tunnel splitting is the dominant energy near zero detuning: $2{t}_{E}\gg {E}_{Z}=g\mu B$, the energy difference between $|g,\downarrow \rangle$ and $|e,\uparrow \rangle$ is too large to allow any significant mixing, therefore hot spots are not present. As magnetic field increases toward *B*_*c*_ that satisfies *gμ*_*B*_*B*_*c*_ = 2*t*_*E*_, the energies of state $|g,\downarrow \rangle$ and $|e,\uparrow \rangle$ become close to each other at zero detuning, and an SOI-induced anti-crossing starts to form between the two states. Consequently a single spin hot spot appears at *B* = *B*_*c*_ and *V*_*d*_ = 0. In a higher magnetic field, the energy of $|g,\downarrow \rangle$ is larger than that of $|e,\uparrow \rangle$ at zero detuning (*V*_*d*_ = 0), so that two anti-crossings form symmetrically on either side of the zero detuning point. The resulting maximum mixture at the anti-crossings produce the two relaxation peaks^55^ in Fig. 4 for a given magnetic field *B* > *B*_*c*_ (One appears at *V*_*d*_ < 0, the other symmetrically at *V*_*d*_ > 0).

Incidentally, the fact that a spin hot spot appears at *B* = *B*_*c*_ can be used to detect the tunneling matrix element *t*_*E*_. A similar method has been used to detect valley splitting in a Si quantum dot^63^.

With the data obtained from Eqs (12) and (13), we also explicitly show the residue coherence after the transport in the lower panel of Fig. 4. The initial state is chosen as a superposition state $|{\psi }_{ini}\rangle =\frac{1}{\sqrt{2}}(|g,\uparrow \rangle +|g,\downarrow \rangle )$, and the evolution time is chosen as 1 ns. The residue coherence (|*ρ*_12_| of final state) is computed from Eq. (14). The figure indicates that in the region *B* < *B*_*c*_, decoherence can be completely neglected. In the region $B\gg {B}_{c}$, when the hot spots appear, the total relaxation is still very small because the peaks of Γ are very sharp and contribute little to the integrated final result. Only in the case of *B* ≈ *B*_*c*_, the relaxation rate Γ peaks in a wide range of *E*-field and causes significant coherence loss.

**Figure 5:**
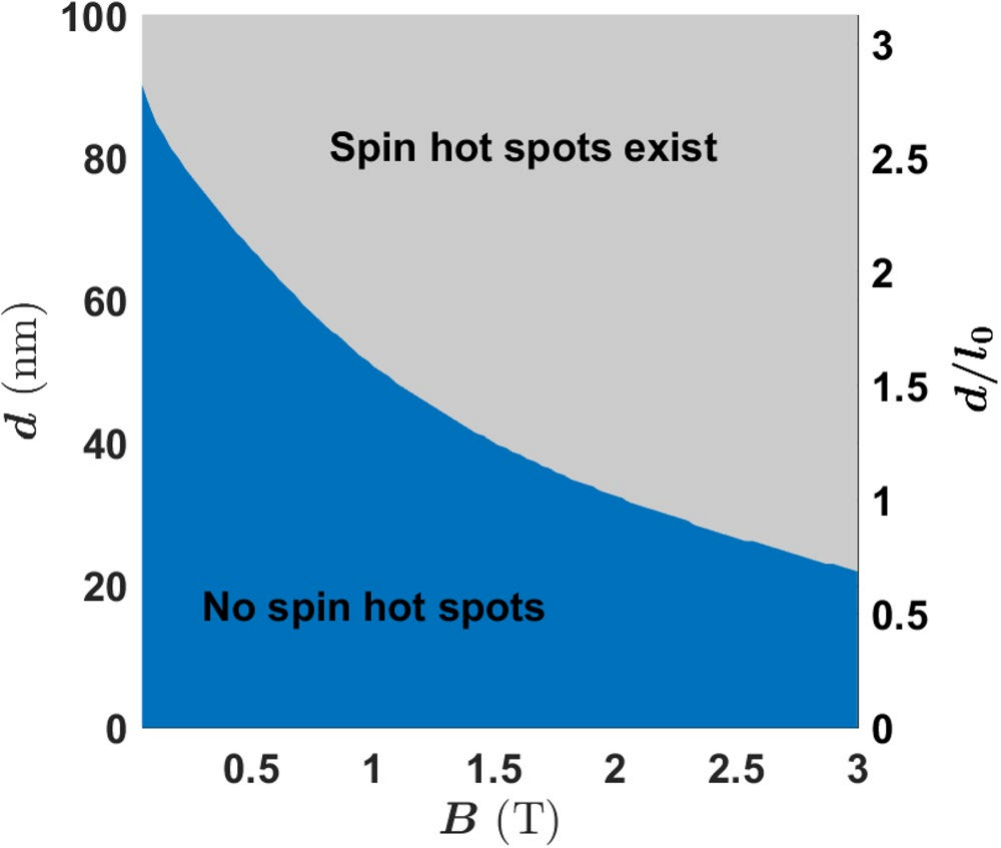
Spin relaxation hot spots in the parameter space of half interdot distance *d* and magnetic field *B*, two important adjustable parameters in experiment. The interdot distance is also measured by the confinement length ${l}_{0}=\sqrt{\hslash /m{\omega }_{0}}=32\,{\rm{nm}}$ as shown in the right-side scale of *y*-axis. Other parameters are the same as in Fig. 4.

In general, the tunnel splitting *t*_*E*_ (as well as *B*_*c*_) is qualitatively related to interdot distance *d*: the larger the *d* is, the higher and wider the tunnel barrier, the lower the *t*_*E*_. We could thus define a safe region in the parameter space expanded by *d* and *B*, where spin hot spots are absent. In Fig. 5 we plot this safe region, in which spin relaxation rate is in the order of Γ ≈ 10^3^ Hz. In the upper-right gray region, hot spots would appear at a certain electric field. Near and at the hot spots, the relaxation rate rapidly increases to the level of Γ ≈ 10^9^ Hz, similar to the relaxation rate of a charge qubit. As mentioned above, the boundary between the two regions is roughly given by the condition ${t}_{E}={E}_{Z}=\frac{1}{2}g{\mu }_{B}B$.

Our results demonstrate that spin relaxation rate is generally quite low in a DQD, in the order of ${\rm{\Gamma }}\lesssim {10}^{3}\,{\rm{Hz}}$ at lower magnetic fields, if we can avoid spin hot spots. Thus, spin relaxation would not usually be an important issue for high fidelity spin transport since tunneling generally happens at the nanosecond time scale. Furthermore, even if an experiment is performed in the “unsafe” region *B* > *B*_*c*_, the accumulated coherence lost is still negligible even though the electron suffers two hot spots during the transport. For example, from the data in lower panel of Fig. 4, the residue coherence is still 99.6% at *B* = 1.3 T. This is because the coherence of electron spin is dependent on a time-integral of the relaxation rate Γ(*t*). Although the relaxation rate Γ(*t*) has two peaks approaching nano-seconds level, the peaks are quite sharp so that the contribution to the integral is still small as long as the sweeping time over the peak is short. Thus we should be able to keep spin relaxation error small as long as the time we sweep through a hot spot is short.

In short, in most cases spin relaxation does not cause any significant negative impact on high-fidelity spin transport, especially if the experimental parameters are tuned to the safe region as suggested in Fig. 5.

### Spin fidelity loss due to systematic errors

In the previous subsection, we investigated the spin transfer fidelity loss due to incoherent processes caused by the external environments, and found that they can be suppressed if certain conditions are met in the transport. In this subsection, we focus on systematic errors that occur inevitably during a finite-speed spin transfer, including non-adiabatic LZ transitions, and a correction to the electron *g*-factor from the double dot potential. We show that these errors can be quite important and have to be accounted for properly, and we discuss how to reduce or eliminate these systematic errors.

#### Landau-Zener transitions

The Landau-Zener transitions occurs when a time-dependent Hamiltonian for a quantum system is swept through a level anti-crossing. Here we study how the fidelity of our spin transport protocol may be affected by such transitions^64–68^.

In an LZ transition, the diabatic transition probability, i.e. the probability that the quantum state does not follow the adiabatic path, is given by^64^

15 $${P}_{D}=\exp \,(-\frac{2\pi {\rm{\Delta }}{E}_{nm}^{2}/\hslash }{d|{E}_{n}-{E}_{m}|/dt}).$$

Here Δ*E*_*nm*_ = (*E*_*n*_ − *E*_*m*_)/2 (at min{*E*_*n*_ − *E*_*m*_}) is half of the energy gap at the anti-crossing point, and *d*|*E*_*n*_ − *E*_*m*_|/*dt* is the time derivative of the gap between the two anti-crossing levels *n* and *m* as the Hamiltonian is linearly swept through the anti-crossing.

As illustrated in Fig. 2(a), in our spin transport protocol the electric field is swept from negative to positive, in the middle of which an orbital-level anti-crossing is formed. If the electric field is increased too fast, unwanted diabiatic transitions will lead to finite probabilities of excitation into excited final states. For example, if the electron is initially in the ground state of the right dot, one possible final excited state is when the electron remains in the right dot ground state and fails to tunnel. Furthermore, Fig. 2(b) shows that at higher magnetic fields, two SOI-induced anti-crossings are also present, giving rise to additional possible diabatic transitions that may or may not be desirable.

Here we focus on possible LZ transitions between orbital states as shown in Fig. 2(a). For our numerical simulation, we choose the energy gap between the ground $|g,\uparrow \rangle$ (or $|g,\downarrow \rangle$) and excited orbital state $|e,\uparrow \rangle$ (or $|e,\downarrow \rangle$) to be about 10 GHz. More precisely, 2Δ*E*_31_ ≈ 2Δ*E*_42_ ≈ 48 *μ*eV. Taking a linearly increasing electric field $E(t)={E}_{0}\frac{t}{T}$ (−*T* ≤ *t* ≤ *T*), *d*|*E*_*n*_ − *E*_*m*_|/*dt* is inversely proportional to the total operation time 2*T*, so that the probability of diabatic transition *P*_*D*_ is an exponentially decaying function of the total operation time 2*T*.

**Figure 6:**
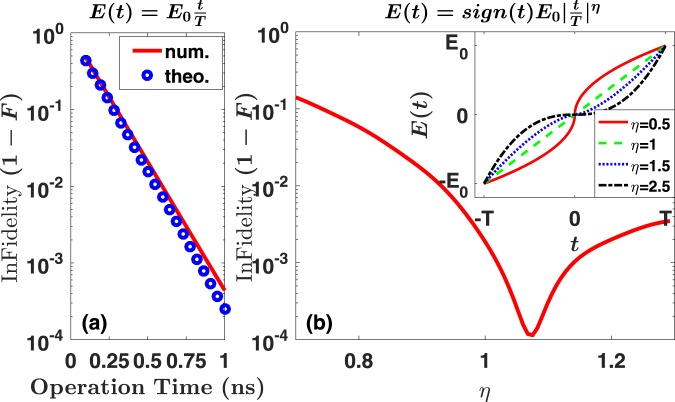
(**a**) Infidelity of spin transport as a function of the total operation time for a linear pulse $E(t)={E}_{0}\frac{t}{T}$. (**b**) Transport infidelity for different power-law exponents of the electric field pulse. The pulse shape is determined by the power *η* as in *E*(*t*) = *E*_0_sign(*t*)|*t*/*T*|^*η*^. For panel (b), the total operation time is fixed at 2*T* = 0.8 ns. For both panels (a and b) *E*_0_ = 4000 V/m, *B* = 1 T.

In Fig. 6(a), we compare the numerical results (red solid line) of the fidelity after the transport defined as *F* = |〈*ψ*_*target*_|*ψ*_*f*_〉|^2^ and the theoretical prediction (blue circles) from Eq. (15). Here, |*ψ*_*f*_〉 represents final state after transport, |*ψ*_*target*_〉 is the designed target state, and *F* is plotted as infidelity 1 − *F*. Suppose we are aiming at transporting the initial state $|g,\uparrow \rangle$ in the left dot, we expect the target state |*ψ*_*target*_〉 should still be $|g,\uparrow \rangle$ in the right dot, namely the evolution follows adiabatic path. The fidelity *F* measures whether final state |*ψ*_*f*_〉 is successfully transported. Notice that the LZ formula (15) agrees quite well with the numerical simulation of the dynamics, even though Eq. (15) is derived for a simple two-level model^64,65^, while our double dot model is complicated by factors including higher orbital states and corrections from SOI. Clearly, the corrections from all the complexities are relatively small and the dynamics of the double dot can be roughly modeled as a two-level (orbital) system. One simple observation we can make here is that in order to achieve high fidelity transport, the time duration of the field-sweep should be sufficiently long to avoid unwanted transitions. For example, for the orbital LZ transition considered here, a total operation time longer than 0.5 ns for a detuning change of 0.74 meV could ensure a 0.99 fidelity.

A linearly varying electric field is far from optimal in ensuring adiabatic electron tunneling between the DQD^69^. As indicated in Eq. (15), one can modify the shape of the detuning voltage pulse in order to keep the system in the ground state. Keeping the total evolution time as a constant, one can design a pulse that changes more slowly near the minimum gap and more quickly away from zero detuning. As an illustration we numerically study several pulses described by $E(t)={E}_{0}{\rm{sign}}(t){|\frac{t}{T}|}^{\eta }$, where time *t* changes from −*T* to *T*, and the function sign(*t*) = 1 for *t* ≥ 0, sign(*t*) = −1 for *t* < 0.

The resulting infidelity 1 − *F* of the evolution is presented in Fig. 6(b). The fidelity is determined by the competition of two factors, sweeping speed near *V*_*d*_ = 0 and time spent near *V*_*d*_ = 0. From the inset of Fig. 6(b), a small *η* has a fast sweeping speed near *V*_*d*_ = 0, but spends less time there. In contrast, a large *η* has a slower sweeping speed near *V*_*d*_ = 0, but the pulse will stay near *V*_*d*_ = 0 for a longer time. The balance of the two factors above leads to the best fidelity, as shown in Fig. 6(b). In other words, a very slow sweep does not lead to higher fidelity of transport. This is in contrast to the case of a single two-level LZ transition. Here we have simply chosen a few power-law functions as an illustration, without any attempt at optimization. There are certainly better pulse shapes to avoid or enhance a transition. One can also design alternative techniques to modify the system evolution. For example, adding extra control pulses can also help remove non-adiabatic contributions, and achieve “shortcuts to adiabaticity”^70,71^.

#### Landau-Zener-Stückelberg interferences

The Landau-Zener-Stückelberg interference could occur when a system Hamiltonian is swept through multiple anti-crossings^64–68^. In this subsection we explore how LZS interference may help maintain spin transfer fidelity at higher magnetic fields.

**Figure 7:**
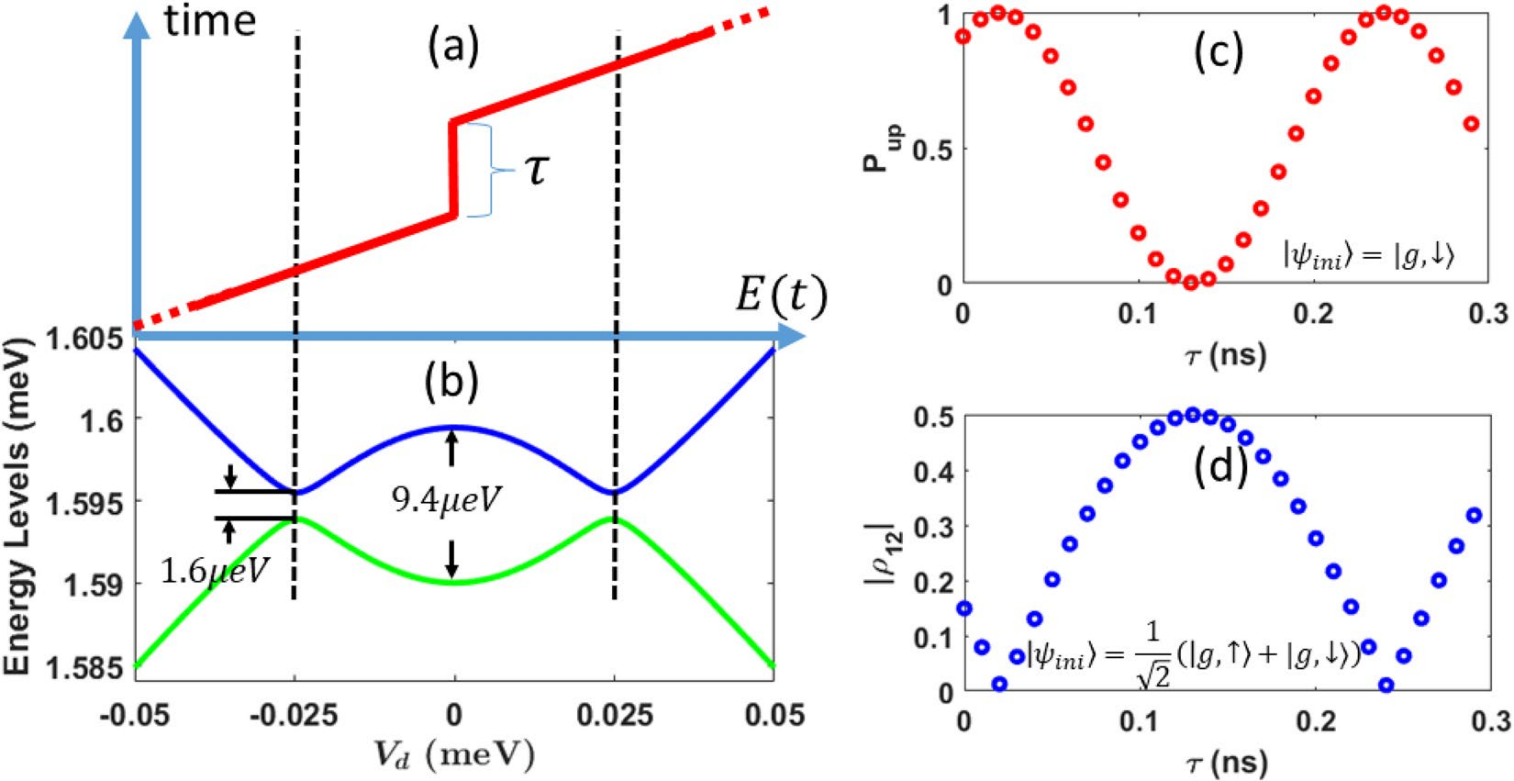
(**a**) Schematic diagram of pulse shape for tuning LZS interference. A plateau is inserted in an otherwise linearly increasing electric field. The duration of the plateau is given by parameter *τ*. (**b**) Energy diagram near zero detuning. The full energy diagram is given in Fig. 2(b) with two other levels. The actual electric field increases from −1500 V/m (*V*_*d*_ = 2*eEd* ≈ −0.14 meV) to 1500 V/m (*V*_*d*_ ≈ 0.14 meV). (**c**) Spin-up probability (*P*_*up*_) in the final state after an extra waiting time *τ* is inserted at the point *V*_*d*_ = 0 in the electric field pulse. The initial state is $|{\psi }_{ini}\rangle =|g,\downarrow \rangle$. (**d**) Matrix elements of reduced density matrix *ρ*. The initial state is $|{\psi }_{ini}\rangle =\frac{1}{\sqrt{2}}(|g,\uparrow \rangle +|g,\downarrow \rangle )$. The applied magnetic field is chosen as *B* = 1.3 T, total operation time without counting *τ* is 2*T* − *τ* = 20 ns.

As shown in Fig. 2(b), when *B* > *B*_*c*_, states $|g,\downarrow \rangle$ and $|e,\uparrow \rangle$ would cross at certain detunings, and spin would mix because of SOI. The resulting anti-crossings mean that unwanted LZ transitions between the two spin states could occur as we sweep the system Hamiltonian through either one of them. The relatively weak SOI in GaAs means that the gap for these anti-crossings are much smaller than the orbital anti-crossing gap. Thus a complete adiabatic evolution requires a much slower sweeping speed. Conversely, a fast passage through these anti-crossings would keep the electron spin unchanged, which is desirable for spin transport. For each of the SOI-induced anti-crossings in Fig. 7(b), the minimum energy gap between *E*_2_ and *E*_3_ is estimated at 2Δ*E*_32_ = 1.6 *μ*eV. The gap is computed by taking the SOI parameters as *α*_*D*_ = 4.5 meV · Å and *α*_*BR*_ = 3.3 meV · Å (see ref.^48^ and experimental references therein). According to Eq. (15), a diabatic transition probability ${P}_{D}\approx \frac{1}{2}$ is possible if we sweep the electric field from −1500 V/m to 1500 V/m (corresponding to *V*_*d*_ = 2*eEd* changing from −0.14 meV to 0.14 meV) in about 20 ns. A numerical simulation confirms this estimate (not shown in the figure).

The presence of two LZ processes near each other opens the possibility of guiding the electron state towards a desired outcome using LZS interference. The energy diagram here is similar to the structure of a two-paths interferometer that is widely used in quantum optics^72–76^. The two energy levels here are similar to the two arms of an interferometer, while the dynamical phase between the energy levels is analogous to the phase difference between two optical paths. These analogies indicate that we should be able to control the output state by manipulating the phase difference between the two energy levels.

One way to control the interference is to add a waiting period *τ* at *E* = 0 to the original linearly-increasing pulse $E(t)={E}_{0}\frac{t}{T}$ in Fig. 7(a). Consider an initial state in the second eigenstate |2〉. After the first LZ process, the state becomes a superposition $|2\rangle \to \sqrt{{P}_{A}}|2\rangle +\sqrt{{P}_{D}}{e}^{i{\varphi }_{0}}|3\rangle$, where *P*_*A*_ and *P*_*D*_ are the adiabatic and diabatic transition probabilities. After the evolution between the two LZ processes, the state becomes $\sqrt{{P}_{A}}|2\rangle +{e}^{i(\varphi +\delta \varphi )}\sqrt{{P}_{D}}|3\rangle$, where *ϕ* is the normal dynamical phase accumulated between the two anti-crossings (including *ϕ*_0_), while *δϕ* is the extra phase that can be controlled by the duration of the wait at *E* = 0. Eventually, after the second LZ process, the state becomes (unnormalized)

16 $$|{\psi }_{out}\rangle =({P}_{A}+{P}_{D}{e}^{i(\varphi +\delta \varphi )})|2\rangle +\sqrt{{P}_{A}{P}_{D}}(1+{e}^{i(\varphi +\delta \varphi )})|3\rangle .$$

In order to obtain an output state |2〉, the extra phase needs to satisfy 1 + *e*^*i*(*ϕ*+*δϕ*)^ = 0. One can also obtain |3〉 by properly choosing a different extra phase in the case *P*_*A*_ = *P*_*D*_. As shown in Fig. 7(c), the output state indeed undergoes the LZS interference and oscillates between spin up and down determined by the extra phase between the two LZ processes. As shown in Fig. 7(c), the probability of obtaining a spin-up state can reach 0 or 1 if an extra phase is properly chosen. Alternatively, this interference may also be employed to achieve controlled spin flip.

The LZS interference can also be used to preserve coherence (superposition). If we transport a superposition state $|{\psi }_{ini}\rangle =\frac{1}{\sqrt{2}}(|g,\uparrow \rangle +|g,\downarrow \rangle )$, namely a superposition of states |1〉 and |2〉, the residue coherence can be preserved by controlling *τ*. Here, we measure the coherence by using the off-diagonal matrix element of the reduced density matrix for spin state *ρ*(*t*) = Tr_*orbital*_(|*ψ*(*x*, *y*, *σ*, *t*〉 〈*ψ*(*x*, *y*, *σ*, *t*)|), which is obtained by tracing out the orbital degree of freedom. In Fig. 7(d), by properly choosing *τ*, the residue coherence of the output state |*ρ*_12_| can be recovered to 0.5 which is the value in initial state. We note that |*ρ*_12_| only reflects amplitude of the coherence, it does not guarantee that the final state is exactly the same as the initial, because there is a relative phase accumulated during the evolution. The final state is supposed to be $|{\psi }_{fin}\rangle =\frac{1}{\sqrt{2}}(|g,\uparrow \rangle +{e}^{i(\varphi +\delta \varphi )}|g,\downarrow \rangle$, where the coherence is preserved but with an extra phase accumulated in the whole evolution. To fully recover the initial state, a phase rotation is required after the transport.

The theoretical analysis above does not include decoherence from the environment. Besides spin relaxation caused by hyperfine interaction or SOI coupling discussed in Sec. 2.2.1, charge noise and orbital relaxation may also lead to errors in an LZS interferometer. For example, charge noise can result in a shift (error) in *V*_*d*_, so that the waiting period *τ* may not be inserted exactly at *V*_*d*_ = 0. The error in energy gap will be reflected in the phase accumulated in *τ* and affect the output state. Fortunately, near *V*_*d*_ = 0, the derivative of the energy gap with respect to the detuning is zero, namely $\frac{d|{E}_{3}-{E}_{2}|}{d{V}_{d}}=0$. Therefore, in the leading order, the variation from *E*-field (reflected on *V*_*d*_) does not affect the energy gap. In other words, the scheme is immune to charge noise in the leading order. All corrections are from higher orders, and can often be neglected.

Another possible error to the LZS interferometer is orbital relaxation. As we have shown in Fig. 4, the spin relaxation at *B* = 1.3 T can only cause a small 0.4% fidelity loss. However, during the evolution, the state could momentarily be excited to higher orbital states, so that orbital relaxation could occur with a higher relaxation rate in the period *τ*. According to our calculation (also in ref.^48^) with the method used in Sec. 2.2.1, the orbital relaxation rate around *B* = 1.3 T is about Γ = 5 × 10^8^ s^−1^, in a waiting time *τ* = 0.1 ns, population loss can be as high as ~2.5%. The best way to reduce the error caused by orbital relaxation is to reduce the excitation by sweeping the detuning more slowly. One could also potentially employ error correction schemes such as feedback control^77^.

The interference pattern in Fig. 7(c) contains useful information about the DQD. Specific to our numerical results, the period of the spin state oscillation is roughly Δ*τ* = 0.22 ns, which indicates that the energy splitting between the 2nd and the 3rd level at zero detuning should be (*E*_3_ − *E*_2_)|_*E*=0_ = 9.4 *μ*eV, since the additional phase difference is given by *δϕ* = (*E*_3_ − *E*_2_)Δ*τ*/ℏ. From the numerical result shown in Fig. 7(b), the zero-detuning energy gap is indeed close to the value predicted from the interference pattern. An accurate measurement of this energy splitting thus gives further information on the tunnel barrier between the two dots.

#### Effective *g*-factor and dynamical phase

In the last two subsections, we investigated gate errors due to LZ transitions. These are essentially population errors from unwanted transitions between the electron spin states. Besides such population errors, phase error could also be an important issue in high-fidelity electron transport, when a superposition state is transferred. In this subsection we investigate a correction to the electron *g*-factor due to the double dot confinement potential, and show that this correction leads to a significant modification to the dynamical phase of the spin, and has to be accounted for properly.

Typically, an electron spin qubit is only viable when its orbital degree of freedom is frozen. This means that the electron should always be in the ground orbital state. During spin transport, the electron should also remain in the instantaneous ground orbital states. This adiabaticity in the orbital motion is maintained by keeping a sweeping rate that is much lower than the orbital excitation energy. However, for high-fidelity spin transfer, freezing of orbital degree of freedom is only a necessary, but not sufficient, condition, especially when a superpositioned spin state is transferred. This is because in a finite magnetic field, a dynamical phase accumulates between the two spin eigenstates. The phase is determined by the electron *g*-factor, which is in turn determined by the full spectrum of the electron and will be modified when the electron confinement potential changes from a single dot to a double dot. Below we study how significant this effect could be in our spin transfer protocol.

The effective *g*-factor for the electron is based on the energy difference between states $|g,\uparrow \rangle$ and $|g,\downarrow \rangle$:

17 $${g}_{eff}=({\varepsilon }_{g,\downarrow }-{\varepsilon }_{g,\uparrow })/{\mu }_{B}B.$$

Without SOI, orbital motion and spin evolve in their own Hilbert sub-space separately, so that the energy difference above is exactly the Zeeman energy *gμ*_*B*_*B*, and *g*_*eff*_ is equal to the bulk value *g*. However, with SOI in a QD or DQD, both ${\varepsilon }_{g,\downarrow }$ and ${\varepsilon }_{g,\uparrow }$ are modified slightly, and the effective *g*-factor also deviate slightly from the bulk value.

**Figure 8:**
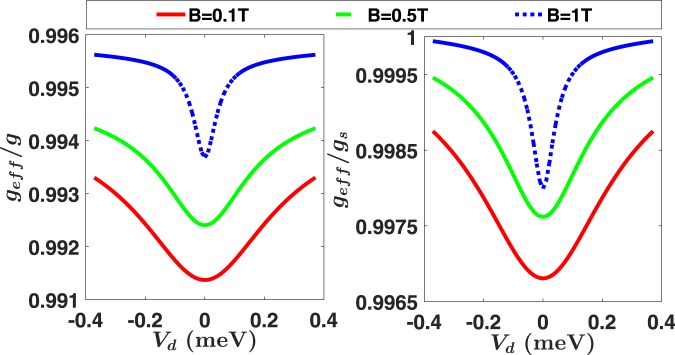
The DQD effective *g*-factor *g*_*eff*_ as a function of the applied electric and magnetic field. The left panel gives the ratio of *g*_*eff*_ over the bulk value, while the right panel is the ratio of *g*_*eff*_ over the effective *g*-factor *g*_*s*_ of a single dot.

In Fig. 8 we plot *g*_*eff*_ as a function of the interdot detuning *V*_*d*_ = 2*eEd*. In the left panel *g*_*eff*_ is normalized against the bulk *g*-factor, while in the right panel it is normalized against the single-dot effective *g*-factor *g*_*s*_. Figure 8 clearly shows that *g*_*eff*_ depends on both the applied magnetic field and the electric field/interdot detuning, and the corrections are the largest at the zero-detuning point. Quantitatively, the correction on the *g*-factor is smaller than 1% in the whole parameter space. Considering that the initial state in a transport experiment is always prepared in the lowest-energy orbital state in a single dot, the results from right panel should be more directly relevant in evaluating the effects of a double dot potential during the spin transport.

The enhancement of the correction on the electron *g*-factor near zero detuning is due to the more even distribution of the orbital wave function across the two dots. Take the spin-orbit Hamiltonian *H*_*SO*_ as a perturbation to *H* = *T* + *V*_0_ + *H*_*Z*_ + *H*_*E*_, the second-order perturbation gives a correction on the *i*^*th*^ energy level $\delta {E}_{i}\sim -\,({\alpha }_{D}^{2}-{\alpha }_{BR}^{2})$ $\mathrm{[1}-\langle i|{\sigma }_{z}(x{\pi }_{y}-y{\pi }_{x})|i\rangle ]$. Mathematically, a wave function distributed more evenly in the two dots has a larger mean value of $\langle i|{\sigma }_{z}(x{\pi }_{y}-y{\pi }_{x})|i\rangle$, leading to a stronger correction on the *g*-factor. Mathematically, in an even double dot the first excited orbital state is much closer to the ground state so that it can give larger contribution to the modification of the *g*-factor.

Any change in the electron *g*-factor would add to the dynamical phase between its two spin orientations. While such a modification does not matter to a spin eigenstate, it could be significant for a superposed state. Consider transporting a superposed state $|g,\uparrow \rangle +|g,\downarrow \rangle$, a phase factor would appear in the final state $|g,\uparrow \rangle +{e}^{i{\rm{\Phi }}}|g,\downarrow \rangle$, where

18 $${\rm{\Phi }}={\int }_{0}^{T}\,\frac{1}{\hslash }{g}_{eff}(\tau ){\mu }_{B}Bd\tau .$$

Here, *g*_*eff*_ (*τ*) is a time-dependent function because *E*(*τ*) changes with time and *g*_*eff*_ depends on *E*. This phase Φ has to be tracked accurately in order to maintain high fidelity of the spin state. Clearly, if one was to use the bulk |*g*| = 0.44 or the single-dot *g*-factor *g*_*s*_ to calculate Φ, the phase information becomes inexact. We can define a phase error as ${{\rm{\Phi }}}_{error}={\int }_{0}^{T}\,\frac{1}{\hslash }\,[{g}_{eff}(\tau )-g]\,{\mu }_{B}Bd\tau$, where *g* = *g*_bulk_ or *g* = *g*_*s*_. Our numerical results show that the accumulated phase error could be non-negligible even though the correction on *g*_*eff*_ is always smaller than 1%, since Φ_*error*_ is an integration over time. For example, the time average of *g*_*eff*_ at *B* = 1T is about ${\bar{g}}_{eff}\approx 0.999{g}_{s}$ (compared to single-dot *g*_*s*_) or ${\bar{g}}_{eff}\approx 0.995g$ (compared to bulk *g*). If we use the bulk value *g* to estimate the phase, a 10 ns operation time will cause a phase error of 0.62*π*. If we use the single dot value *g*_*s*_ to estimate the phase, the error can still reach 0.12*π* when the operation time is 10 ns. Therefore, in the calculation of dynamical phase, the double dot corrections on the *g*-factor must be taken into consideration.

Equation (18) is based on the assumption that the time evolution during spin transport is slow enough so that *g*-factor is always well defined. Corrections with a geometric interpretation due to non-adiabatic evolution could become more significant as the speed of the evolution increases. A more in-depth study of this limit remains open.

In summary, a double-dot-modified *g*-factor can cause significant corrections to the dynamical phase of a superposed spin state, and need to be addressed when transporting a coherent spin state. The modification to the dynamical phase is systematic, and is completely determined by the SOI coupling strength, the double dot potential, and the detuning sweeping pulse shape. The correction from the double dot potential must be taken into consideration when calculating the overall dynamical phase. Even using the value of the *g*-factor for a single dot will still lead to a notable error in calculating the dynamical phase, while using the bulk value causes a much larger error.

Similar to the population errors due to LZ transitions, and different from the incoherent error due to relaxation and dephasing, the phase error here is a deterministic effect and can be predicted accurately if one has enough information of the system parameters. Therefore, it can be eliminated precisely by performing a phase rotation based on an accurate calculation or measurement of the dynamical phase.

Experimentally, one can calibrate the dynamical phase empirically by transporting a known superposed state (e.g., with an initial phase Φ_*ini*_) with a given pulse sequence under desired conditions such as a particular applied field. By measuring the phase of the final state (e.g., with phase Φ_*fin*_), the accumulated phase shift can be determined. For an unknown state transporting under the same parameters and pulse sequence, one can then perform a phase shift (Φ_*fin*_ − Φ_*ini*_) to eliminate the accumulated phase during the transport.

## Discussion

In this paper, we have investigated how to maintain high fidelity when transporting an electron spin qubit in a GaAs DQD. We analyze three factors potentially affecting the transport fidelity, the spin relaxation and dephasing, the non-adiabatic transitions in an LZ process, and the double-dot corrections to the electron *g*-factor.

It is known that the SOI-induced level mixing between different spin states can produce spin hot spots^48,55^. Here we show clearly where these hot spots are, and calculate their accumulated impact on the fidelity loss in a transport process. Using the guideline *gμ*_*B*_*B* < *t*_*E*_, we identify a safe region in the parameter space to avoid spin hot spots. Furthermore, even in the unsafe region $g{\mu }_{B}B\gg {t}_{E}$, where spin hot spots are present, we find that the accumulated fidelity loss can still be negligible as long as the sweeping time over the peak is sufficiently short.

We also study spin transfer fidelity loss caused by the random Overhauser field from hyperfine interaction. We estimate the fidelity loss caused by both transverse and longitudinal nuclear fields. For a typical strength of transverse nuclear filed in realistic experiments *B*_*nuc*_ ~ 2 − 6 mT, the fidelity loss caused by spin precession is negligible at a typical external magnetic field *B* ~ 1 T. On the other hand, a random longitudinal nuclear field causes inhomogeneous broadening, and can be corrected by spin echo. Furthermore, electron motion can lead to motional narrowing, which leads to suppression of inhomogeneous broadening. We would also like to mention here that inhomogeneous broadening can be further reduced by engineering of the nuclear environment^78,79^. For example, by using real-time Hamiltonian estimation, the inhomogeneous broadened dephasing time can exceed 2 *μ*s in a GaAs quantum dot^80^. The errors can be also suppressed by dynamical decoupling even if the detailed information about the nuclear field is unknown^54^, and the efficiency of the error correction can be further improved by optimizing the pulse sequence^7,81^.

In the regime where spin relaxation effect is minimized, we find that spin transfer fidelity can still be reduced by coherent processes such as non-adiabatic spin flip due to Landau-Zener transitions. We demonstrate how pulse shaping of the detuning pulse sequence can help increase the transfer fidelity. We also propose a scheme to achieve high-speed electron transport through Landau-Zener-Stückelberg interference, and show that such a scheme can be used to measure the tunnel barrier between the two dots as well.

Last but not least, we study an important correction to the electron *g*-factor by SOI and the double dot potential. We point out that while this correction in *g*-factor is always under 1%, missing the correction can cause a significant error in the tracking of the phase difference between spin up and down states. Consequently, if a superposed spin state is to be transferred with high fidelity, the modified dynamical phase has to be accurately accounted for.

Spin transport in other semiconductor materials should have qualitatively similar behaviors, though quantitatively the differences from GaAs could be significant. For example, InSb has much stronger SOI as compared to GaAs, and it has been suggested that electron transport could be important for testing and manipulating Majorana fermion excitations in InSb nanowires^38,39^. In InSb the energy correction from SOI would be more significant, which means larger anti-crossing gaps and larger corrections on the *g*-factor.

Silicon quantum dots^63,82,83^ have a much cleaner nuclear environment, which may provide super long coherence time. However, silicon quantum dots present another interesting challenge to spin transport. The nearly degenerate valley states in the conduction band could introduce significant additional complexities into spin transport. Specifically, spin-valley mixing can cause a new type of spin relaxation^63^, while the extra valley degree of freedom can produce additional anti-crossings and interference between these anti-crossings^34^. Such new features and challenges will be investigated elsewhere.

## Methods

### Derivation of the spin master equation under SOI and its formal solution

The spin relaxation caused by SOI via electron-phonon interaction can be modeled a an amplitude damping process^60^, for which we have already computed the time-dependent damping rate Γ(*t*). In the “quantum jump” point of view^61^, the impact from the effective environment can be roughly modeled as damping from higher state to the ground state with probability Γ(*t*). For a single spin, if $|\psi ({t}_{0})\rangle =|\,\downarrow \,\rangle$, then

19 $$|\psi ({t}_{0}+dt)\rangle \to (\begin{array}{ll}|\,\uparrow \,\rangle  & {\rm{with}}\,{\rm{probability}}\,{\rm{\Gamma }}({t}_{0}),\\ |\,\downarrow \,\rangle  & {\rm{with}}\,{\rm{probability}}\,1-{\rm{\Gamma }}({t}_{0}).\end{array}$$

Such a process can be equivalently described by the master equation^61^

20 $$\frac{d}{dt}\rho =-\,\frac{i}{\hslash }[{H}_{Z},\rho ]-\frac{{\rm{\Gamma }}(t)}{2}({\sigma }_{+}{\sigma }_{-}\rho +\rho {\sigma }_{+}{\sigma }_{-})+{\rm{\Gamma }}(t){\sigma }_{-}\rho {\sigma }_{+}.$$

For the off-diagonal element *ρ*_12_(*t*), the dynamical equation is

21 $$\frac{d}{dt}{\rho }_{12}(t)=-\,[i\omega +\frac{{\rm{\Gamma }}(t)}{2}]{\rho }_{12}(t),$$

where *ω* = *gμ*_*B*_*B*. The formal solution of *ρ*_12_(*t*) is

22 $${\rho }_{12}(t)={\rho }_{12}(0)\,\exp \,[-i\omega -{\int }_{0}^{t}\,ds\frac{{\rm{\Gamma }}(s)}{2}].$$

This approach of investigating relaxation is also used in a recent paper^84^.
